# Molecular characterization of *Polychromophilus* parasites of *Scotophilus kuhlii* bats in Thailand

**DOI:** 10.1017/S003118202000222X

**Published:** 2021-04

**Authors:** Chatree Chumnandee, Nawarat Pha-obnga, Oskar Werb, Kai Matuschewski, Juliane Schaer

**Affiliations:** 1 Department of Animal Science, Faculty of Agriculture and Technology, Nakhon Phanom University, Nakhon Phanom 48000, Thailand; 2 Department of Molecular Parasitology, Institute of Biology, Humboldt University, 10115 Berlin, Germany

**Keywords:** Haemosporida, *Polychromophilus*, *Scotophilus*, Thailand

## Abstract

Parasites of the haemosporidian genus *Polychromophilus* have exclusively been described in bats. These parasites belong to the diverse group of malaria parasites, and *Polychromophilus* presents the only haemosporidian taxon that infects mammalian hosts in tropical as well as in temperate climate zones. This study provides the first information of *Polychromophilus* parasites in the lesser Asiatic yellow bat (*Scotophilus kuhlii*) in Thailand, a common vespertilionid bat species distributed in South and Southeast Asia. The gametocyte blood stages of the parasites could not be assigned to a described morphospecies and molecular analysis revealed that these parasites might represent a distinct *Polychromophilus* species. In contrast to *Plasmodium* species, *Polychromophilus* parasites do not multiply in red blood cells and, thus, do not cause the clinical symptoms of malaria. Parasitological and molecular investigation of haemosporidian parasites of wildlife, such as the neglected genus *Polychromophilus*, will contribute to a better understanding of the evolution of malaria parasites.

## Introduction

Malaria parasites (order Haemosporida) infect birds, squamates, chelonians and several groups of mammals, including humans, and are transmitted by different groups of haematophagous dipterans (Garnham, 1966). The human-infecting parasite species belong to the genus *Plasmodium*, which is only one out of at least 15 genera that together comprise over 500 haemosporidian species. Parasites of this diverse group differ in host specificities, adaptations and their life cycles (Garnham, 1966). For instance, all haemosporidian genera, except *Plasmodium*, lack the distinct replication phase inside red blood cells, which is the exclusive cause of clinical symptoms of malaria. Therefore, studying the diversity and evolution of the entire haemosporidian parasite group will contribute to our understanding of the important malaria disease in humans (Galen *et al*., 2018).

Parasites of the haemosporidian genus *Polychromophilus* are transmitted by ectoparasitic highly specialized nycteribiid flies and have exclusively been described in bats (Dionisi, 1898; Garnham, 1966, 1973; Witsenburg *et al*., 2012). *Polychromophilus* presents the only haemosporidian taxon that infects mammalian hosts in tropical as well as in temperate climate zones. These parasites are common in bats in Europe and in the tropical regions of Africa, Asia, Australia and South America (e.g. Garnham, 1966; Perkins and Schaer, 2016). Even though *Polychromophilus* parasites are widespread and common, only five morphospecies have been formally described to date. *Polychromophilus murinus* has been mainly reported in bats of the family Vespertilionidae and *Polychromophilus melanipherus* in bats of the family Miniopteridae (e.g. Garnham, 1966; Gardner and Molyneux, 1988). The species *Polychromophilus corradetti* and *Polychromophilus adami* have been described from African *Miniopterus* species (Landau *et al*., 1980). The description of *Polychromophilus deanei* from *Myotis nigricans* (Vespertilionidae) in Brazil, and three other records of *Polychromophilus* from bats in Brazil and Southern USA provided evidence of chiropteran haemosporidian parasites in the New World (Wood, 1952; Deane and Deane, 1961; Garnham *et al*., 1971; Foster, 1979). Several phylogenetic studies have confirmed that *P. murinus* and *P. melanipherus* comprise distinct species (e.g. Megali *et al*., 2011; Witsenburg *et al*., 2012), the latter possibly representing a species complex (Duval *et al*., 2012). In molecular phylogenies, sequences from *Polychromophilus* of *M. nigricans* from Panama, which might represent *P. deanei*, group closely with *P. murinus* parasite sequences (Borner *et al*., 2016). The remaining two morphospecies have not been included in phylogenetic analyses yet, however *Polychromophilus* sequences sampled from the African *Miniopterus* host species of *P. corradetti* and *P. adami* grouped within the *P. melanipherus* clade (Duval *et al*., 2012; Rosskopf *et al*., 2019).

Very few studies have focused on morphological or molecular investigations of *Polychromophilus* parasites in Asia. Two morphological studies described *Polychromophilus* from hipposiderid bat species in Thailand and Malaysia (Eyles *et al*., 1962; Landau *et al*., 1984). One molecular study published a *Polychromophilus* sequence from the vespertilionid bat *Kerivoula hardwickii* in Cambodia and a recent study published two short cytochrome *b* sequences for *P. murinus* and *P. melanipherus* from *Myotis siligorensis* (Vespertilionidae) and *Taphozous melanopogon* (Emballonuridae) in Thailand (Duval *et al*., 2007; Arnuphapprasert *et al*., 2020). Here, data are presented from molecular investigations of *Polychromophilus* infections in the lesser Asiatic yellow bat (*Scotophilus kuhlii*) in Thailand that were originally reported as unidentified haemosporidian parasites in a preliminary morphological study on white blood cell counts of *S. kuhlii* (Chumnandee and Pha-obnga, 2018) and add important information to the phylogeny of these neglected parasites.

## Materials and methods

Bats were captured in April 2018 in the Muang district in the Nakhon Phanom province in Thailand (17°24′38.92″N and 104°46′42.82″E) using standard mist nets. A total of 44 bats were captured from the same colony. Standard morphological measurements were taken for each bat and the identification keys of Duengkae (2007) and Srinivasulu *et al*. (2010) were used for species identification. Bats were kept individually in cotton bags. Blood sampling followed approved animal care protocols and comprised 0.6–1.0% body mass of blood (e.g. 6–19 *μ*L g^−1^) per bat (e.g. Predict One Health Consortium, 2013). The blood samples were used to prepare two thin blood smears and to preserve blood on DNA FTA cards. Bats were released at the capture side, once they had fully recovered. The thin blood smears were fixed and stained with Wright-Giemsa (following Paksuz *et al*., 2009). Slides were thoroughly scanned by light microscopy with a magnification of ×1000 using oil immersion. The morphology of the blood stages of the parasites was compared to original species descriptions. Parasitaemia was calculated as the percentage of parasite-infected erythrocytes in the total number of erythrocytes (total number of parasites/products of mean number of erythrocytes per field × number of counted fields). The mean number of erythrocytes per field was determined by counting three fields and the number of parasites was recorded in 50 fields (fields with comparable erythrocyte density).

Whole genomic DNA was extracted from blood dots on DNA FTA cards using the DNeasy extraction kit (Qiagen). Two mitochondrial genes of the bats were amplified and sequenced to verify the morphological taxonomic identification of the bats (cytochrome *b*, *cytb* and part of the NADH dehydrogenase subunit 1, *ND1*) (Table S1**)**. Sequences were compared to references in GenBank using the NCBI BLAST tool (e.g. Johnson *et al*., 2008). Four genes from the three genomes of the parasites were amplified, the mitochondrial cytochrome *b* (cytb) and cytochrome oxidase I (*cox1*), the nuclear elongation factor 2 (*EF2*) and the apicoplast caseinolytic protease (*clpC*) using established protocols and primers (e.g. Martinsen *et al*., 2008; Schaer *et al*., 2013) (Table S1; see Fig. S1 for primer locations). PCR products were sequenced in both directions and run on an ABI-373 sequencer (accession numbers listed in Table S2). The sequence data were combined with corresponding gene sequences of representatives of the major haemosporidian taxa that were obtained from GenBank (Table S2). Phylogenetic analysis of the concatenated dataset (total of 2793 bp: 978 bp of *cytb*, 957 bp of *cox1*, 483 bp of *clpC*, 375 bp of *EF2*) was carried out with PartitionFinder v.2 (Lanfear *et al*., 2017) and MrBayes v3.2.7 (Ronquist *et al*., 2012) *via* the CIPRES Portal (Miller *et al*., 2010) (Table S3). Bayesian inference methods were carried out with two runs of four chains (heated = 3, cold = 1, temperature = 0.01) each for 10 million generations. The first 25% of trees were discarded as burn-in. Tracer v1.6 was used to evaluate the mixing and convergence of runs and effective sample sizes (EES > 500) (Rambaut *et al*., 2014). Trees were visualized with FigTree v1.4.4.

## Results

The survey of haemosporidian parasites in a colony of *S. kuhlii* bats identified *Polychromophilus* infections in five out of 44 bat individuals (prevalence = 11%). This is the first host record for the vespertilionid bat genus *Scotophilus* and for the species *S. kuhlii* for infections with *Polychromophilus* parasites. The morphological bat species identifications were confirmed with molecular barcoding. The whole mitochondrial cytochrome *b* was sequenced, which featured a 99.7% nucleotide identity with the *S. kuhlii* reference sequences (e.g. EU750921) in GenBank. In addition, 928 bp of the mitochondrial NADH dehydrogenase subunit 1 were sequenced and nucleotide identity with an *S. kuhlii* reference sequence (AB079818) was 98.9% (accession numbers listed in Table S3).

**Fig. 1.**
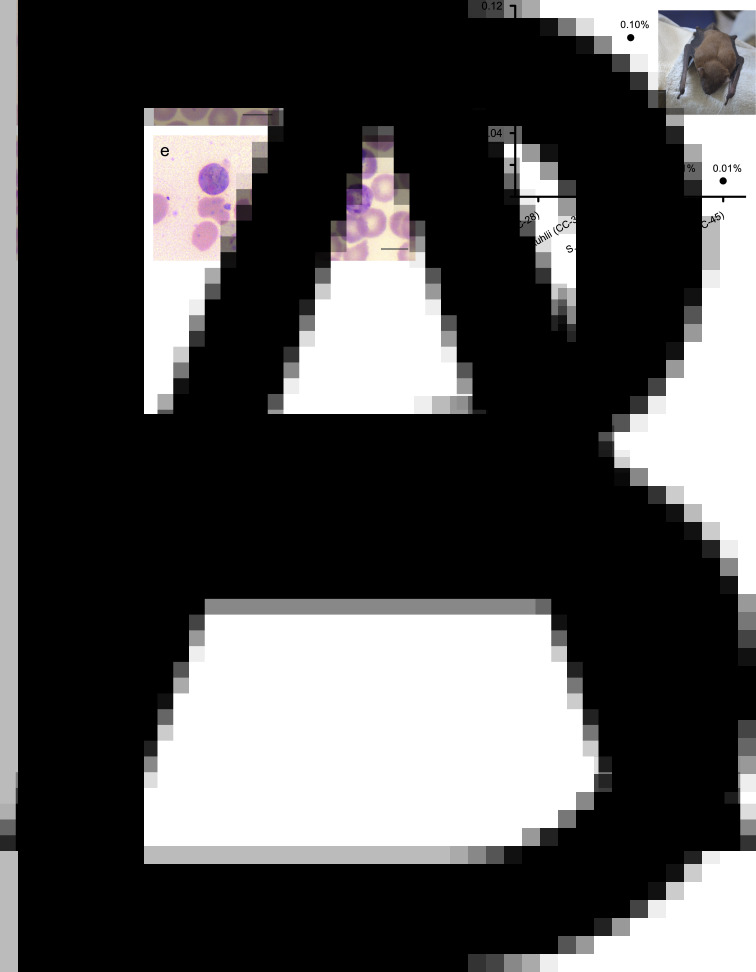
(A) Representative Giemsa-stained micrographs of gametocyte blood stages of *Polychromophilus* parasites from *Scotophilus kuhlii* in Thailand (a, c–d from bat sample CC-33; b, f from bat sample CC-28). Size bars = 5 *μ*m, magnification = 1000×. (a) Immature gametocyte with pale cytoplasm and a peripheral purple nucleus. (b–f) Mature gametocytes that entirely occupy and slightly enlarge the host erythrocytes. The malaria pigment hemozoin is visible as fine dark grains scattered throughout the cytoplasm. (b–c) Male microgametocytes with the cytoplasm in a characteristic light pink colour and the small nucleus in a slightly darker pink. (d–f) Female macrogametocytes with a purple-blue cytoplasm and small nuclei in pink. (B) Parasitaemia in %. Parasitaemia values in the five infected *S. kuhlii* ranged between 0.01 and 0.1% (prevalence of 11%, 5/44 *S. kuhlii* infected). Inserted photograph of *S. kuhlii.*

The blood stages of *Polychromophilus* parasites are limited to gametocytes and the morphology corresponds to the description of *Polychromophilus* parasites of vespertilionid hosts. In Giemsa-stained blood smears, the immature parasites feature a pale cytoplasm and the nucleus is located peripherally and stains purple (Fig. 1A a). When mature, the gametocytes fill the host cell completely and cause a slight enlargement of the erythrocyte. Fine hemozoin pigment grains are scattered in the cytoplasm, a characteristic that is attributed to *P. murinus* (Fig. 1A b–f). In marked contrast, the pigment of *P. melanipherus* is much larger and coarse-grained. The male microgametocytes feature a light pink-stained cytoplasm (Fig. 1A b–c), whereas the female macrogametocytes stain purple-blue (Fig. 1A d–f), both exhibiting a small distinct pink-staining nucleus that is placed eccentrically. The morphology of the gametocyte stages did not allow a clear assignment to any described morphospecies.

The mean *Polychromophilus* gametocytaemia in the blood smear-positive samples was 0.05% (minimum of 0.01% and maximum of 0.1%) (Fig. 1B).

Sanger sequencing revealed that the parasite *cytb* nucleotide sequences were identical, while we noted that the *cox1* sequences in one out of five samples differed by one base. Hence, the five *S. kuhlii* individuals were infected with one cytochrome *b* haplotype and two cytochrome oxidase 1 haplotypes of the same *Polychromophilus* species.

**Fig. 2.**
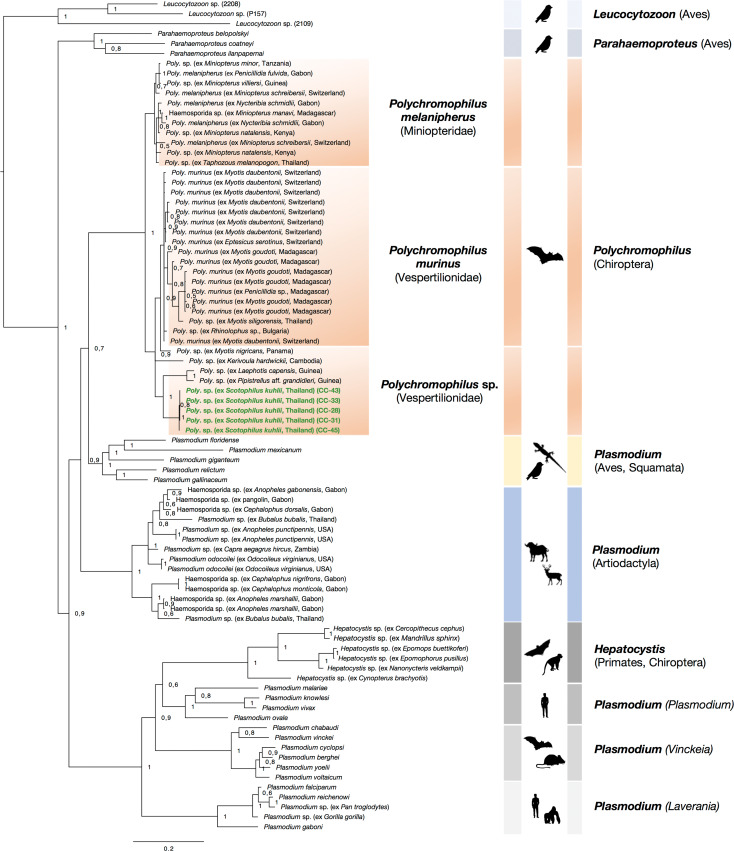
Bayesian analysis of *Polychromophilus* parasites (highlighted in orange) and selected haemosporidian taxa. Posterior probability values are given. The concatenated phylogeny was conducted *via* analysis of four genes, the mitochondrial cytochrome *b* and cytochrome oxidase I, the nuclear elongation factor 2, and the apicoplast caseinolytic protease. Note that the placement of *Polychromophilus* parasites as sister clade to a group that contains the lizard and bird *Plasmodium* species (highlighted in yellow) instead of the ungulate *Plasmodium* species (highlighted in blue) (as confirmed in Galen *et al*., 2018) can be partly attributed to missing *clpC* and *EF2* sequences in *Polychromophilus* reference samples. The monophyletic clade (posterior probability of 1) of *Polychromophilus* parasites comprises two distinctive subclades. One subclade includes all *Polychromophilus melanipherus* sequences isolated from *Miniopterus* bats. A second subclade encompasses all sequences of *Polychromophilus* parasites isolated from vespertilionid bats, including *Polychromophilus murinus* and *Polychromophilus* sp. The ‘Vespertilionidae’ subclade is again divided into two groups, one contains all ‘*P. murinus*’ sequences and the other distinct group comprises the sequences of this study, *Polychromophilus* sequences of *Scotophilus kuhlii* from Thailand (samples highlighted in green), and two *Polychromophilus* sequences isolated from *Pipistrellus* aff. *grandidieri* and *Laephotis capensis* (as *Neoromicia capensis*) in Guinea (Schaer *et al*., 2013). The phylogenetic placement of the Guinean *Polychromophilus* and of the *Polychromophilus* parasites of *Scotophilus kuhlii* bats as distinct from the *P. melanipherus* and *P. murinus* could indicate that they present separate species.

The three-genome phylogeny of *Polychromophilus* in the context of the major haemosporidian parasite clades recovered the *Polychromophilus* parasites (Fig. 2, highlighted in orange) as sister clade to a group that contains the lizard and bird *Plasmodium* species (highlighted in yellow), confirming previous studies that showed a distant relationship of *Polychromophilus* parasites to *Plasmodium* and *Hepatocystis* of mammalian hosts (highlighted in grey) (Fig. 2). Together, they group with the *Plasmodium* species of ungulates (Fig. 2, highlighted in blue). All *Polychromophilus* sequences group into one monophyletic clade (posterior probability of 1) that contains two main subclades. The first distinct subclade comprises all sequences of *P. melanipherus* of *Miniopterus* bat hosts (and one parasite sequence of a *Taphozous* bat host) and the second subclade exclusively includes sequences of *Polychromophilus* parasites of vespertilionid (and one rhinolophid) bat species, confirming a clear separation of parasites of miniopterid and vespertilionid hosts. The second subclade contains *P. murinus* sequences from bats in Europe, Madagascar and Thailand and one sequence that is basal to *P. murinus*, a sample from *M. nigricans* from Panama. The placement of the sample from *K. hardwickii* from Cambodia could not be resolved. The other subclade that is separated from the ‘*P. murinus*’ clade contains the sequences of *Polychromophilus* of *S. kuhlii* from Thailand (Fig. 2, highlighted in green) and two parasite samples from *Pipistrellus* aff. *grandidieri* and *Laephotis capensis* from Guinea.

## Discussion

This study provides the first information on haemosporidian parasites in the bat species *S. kuhlii* in Thailand. The morphology of the blood stages and the phylogenetic analysis identify the parasites as belonging to the genus *Polychromophilus*. The infections featured low overall parasitaemias as reported from other *Polychromophilus* infections (e.g. Rosskopf *et al*., 2019). The three-genome phylogeny confirms a clear separation of *Polychromophilus* parasites of *Miniopterus* bat species and of vespertilionid bat species, the latter including the parasites of *S. kuhlii*. The phylogenetic analysis recovered the *Polychromophilus* parasites as sister clade to a group that contains the lizard and bird *Plasmodium* species, as shown before (Witsenburg *et al*., 2012). However, the most comprehensive phylogeny based on multiple nuclear markers clearly placed *Polychromophilus* as sister clade to the ungulate *Plasmodium* species (Galen *et al*., 2018). Thus, the placement of *Polychromophilus* as sister to the avian/lizard *Plasmodium* species in our analysis can likely be attributed to the unavailability of *cox1*, *clpC* and *EF2* sequences for the majority of the *Polychromophilus* references that were included in the analysis (Tables S2 and S3). Genes display different rates and patterns of evolution and analysing genes of the parasites’ three genomes for robust phylogenies of haemosporidian parasites has been established (e.g. Martinsen *et al*., 2008). However, many phylogenetic studies are still limited to the analysis of (rather short) cytochrome *b* sequences.

To date, only four studies have reported *Polychromophilus* parasites from Asian bats. Eyles *et al*. (1962) reported *Polychromophilus* parasites in the bat species *Hipposideros bicolor* in Malaysia and described the gametocytes as oval in shape, with clear-cut borders and that the parasites only partially occupy the host erythrocytes (Eyles *et al*., 1962). Another morphological study described *Polychromophilus* from *Hipposideros larvatus* in Thailand (as *Biguetiella minuta* which was considered as a vicariant form of *Bioccala*, a subgenus of *Polychromophilus*) (Landau *et al*., 1984). The gametocytes of the latter were also described as not filling the host cell. Thus, the gametocytes of *Polychromophilus* from hipposiderid hosts differ from the morphology of the mature gametocytes observed in the current study that fill the entire host cells and even slightly enlarge the erythrocytes. The only study that reported *Polychromophilus* from a vespertilionid bat species in Asia is that of Duval *et al*. (2007) that found *K. hardwickii* in Cambodia infected with *Polychromophilus* sp. (Duval *et al*., 2007). In our phylogenetic analysis, the nucleotide sequence of *Polychromophilus* of *K. hardwickii* is separated from *Polychromophilus* of *S. kuhlii*. Therefore, we assume that the *Polychromophilus* parasites of *S. kuhlii* in Thailand do not represent the parasites detected in Asian hipposiderid hosts nor the *Polychromophilus* parasite reported from *K. hardwickii* in Cambodia. The phylogenetic analyses resulted in the placement of *Polychromophilus* of *S. kuhlii* outside the *P. melanipherus* and *P. murinus* clades, which also contain the two recently reported *Polychromophilus* parasites from Thailand (Arnuphapprasert *et al*., 2020). The *Polychromophilus* parasites of *S. kuhlii* form a group with the Guinean *Polychromophilus* parasites that have been suggested to represent a distinct species (Schaer *et al*., 2013; Rosskopf *et al*., 2019). Within this group, the *Polychromophilus* parasites of *S. kuhlii* are clearly separated from the Guinean samples (posterior probability = 1) and might therefore also present a distinct species.

Future morphological studies that investigate the tissue stages and molecular studies of additional *Polychromophilus* parasites of Asian bats are needed to reassess this assumption. The host species *S. kuhlii* is widely distributed in South Asia, southern China and Southeast Asia and is found in primary and secondary habitats, both in rural and urban areas and might represent a species complex (Trujillo *et al*., 2009; Srinivasulu and Srinivasulu, 2019). Systematic sampling of *S. kuhlii* across its distribution range and of other potential vespertilionid bat host species will add important information on the host specificity, the prevalence and nycteribiid vectors of *Polychromophilus* parasites in Asia.

## Data Availability

Nucleotide sequence data reported in this paper are available in the GenBank database under accession nos. MT750305-MT750321.

## Supplementary material

For supplementary material accompanying this paper visit https://doi.org/10.1017/S003118202000222X. click here to view supplementary material
