# Supplementary material for: Molecular characterization of Polychromophilus parasites of Scotophilus kuhlii bats in Thailand
Source: Parasitology. 2020 Dec 1;148(4):495–9. doi: 10.1017/S003118202000222X (PMC7938340; doi:10.1017/S003118202000222X)
Supplement: Supplementary file 1 [file S003118202000222Xsup001.docx]

**SUPPLEMENTAL MATERIAL**

**Molecular characterization of *Polychromophilus* parasites of *Scotophilus kuhlii* bats in Thailand**

Chatree Chumnandee, Nawarat Pha-obnga, Oskar Werb, Kai Matuschewski, Juliane Schaer

**Content:**

- **Supplemental Table S1 and Figure S1**
- **Supplemental Table S2**
- **Supplemental Table S3**

**Chumnandee et al. Supplemental Table S1.**

**Supplemental Table S1:** Nucleotide primers used in this study

| **Gene** | **Primer name** | **Sequence (5´- 3´)^1^** | **Reference** |
| --- | --- | --- | --- |
| ***Cytb*** (*Polychromophilus*) | DW2 | TAATGCCTAGACGTATTCCTGATTATCCAG | Perkins & Schall 2002 |
|  | DW4 | TGTTTGCTTGGGAGCTGTAATCATAATGTG | Perkins & Schall 2002 |
|  | 3932-F | GGGTTATGTATTACCTTGGGGTC | Perkins & Schall 2002 |
|  | 3932-R | GACCCCAAGGTAATACATAACCC | Perkins & Schall 2002 |
| ***Cox1***  (*Polychromophilus*) | Cox1-F | CTATTTATGGTTTTCATTTTTATTTGGTA | Martinsen et al. 2008 |
|  | Cox1-R | AGGAATACGTCTAGGCATTACATTAAATCC | Martinsen et al. 2008 |
|  | Cox-in-F | ATGATATTTACARTTCAYGGWATTATTATG | Martinsen et al. 2008 |
|  | Cox-in-R | GTATTTTCTCGTAATGTTTTACCAAAGAA | Martinsen et al. 2008 |
|  | Cox-mid-F | TTATTCTGGTTTTTTGGTCATCCAG | Martinsen et al. 2008 |
|  | Cox-mid-R | CTGGATGACCAAAAAACCAGAATAA | Martinsen et al. 2008 |
| ***clpC***  (*Polychromophilus*) | Clpc-out-F | AAACTGAATTAGCAAAAATATTA | Martinsen et al. 2008 |
|  | Clpc-out-R | CGWGCWCCATATAAAGGAT | Martinsen et al. 2008 |
|  | Clpc-in-F | GATTTGATATGAGTGAATATATGG | Martinsen et al. 2008 |
|  | Clpc-in-R | CCATATAAAGGATTATAWG | Martinsen et al. 2008 |
| ***EF2***  (*Polychromophilus*) | EF2-F | GTTCGTGAGATCATGAACAAAAC | Schaer et al. 2013 |
|  | EF2-R | CCTTGTAAACCAGAACCAAA | Schaer et al. 2013 |
|  | EF2F (Falk) | CATGGAAAATCAACATTAACAGATTCT | Falk et al. 2015 |
|  | EF2R (Falk) | CAGGATATACTTGAATATCACCCAT | Falk et al. 2015 |
|  | EF2Fin (Falk) | AGACAAGATGAACAAGAAAGATGT | Falk et al. 2015 |
|  | EF2Rin (Falk) | TCACCCATTAATTTATCTGTGTATGT | Falk et al. 2015 |
| ***Cytb***  (*Scotophilus*) | L14724 | CGAAGCTTGATATGAAAAACCATCGTTG | Päabo 1989 |
|  | H15915 | GGAATTCATCTCTCCGGTTTACAAGAC | Irwin et al. 1991 |
| ***ND1***  (*Scotophilus*) | ER65 (PCR primer)* | CCTCGATGTTGGATCAGG | Ruedi & Mayer 2001 |
|  | ER66 (PCR primer)* | GTATGGGCCCGATAGCTT | Ruedi & Mayer 2001 |

Cytb, cytochrome b; Cox1, cytochrome oxidase I; *clpC*, apicoplast caseinolytic protease; EF2, nuclear elongation factor 2; *sequenced with internal primers as described in Petit, 1998; ^1^ for position in target gene see Figure S1.

**References**

**Falk BG, Glor RE, Perkins SL** (2015) Clonal reproduction shapes evolution in the lizard malaria parasite *Plasmodium floridense*. Evolution **69**, 1584-1596.

**Irwin DM, Kocher TD, Wilson AC** (1991) Evolution of the Cytochrome *b* gene of mammals. *Journal of Molecular Evolution* **32**, 128 – 144.

**Martinsen ES, Perkins SL and Schall J** (2008) A three-genome phylogeny of malaria parasites (*Plasmodium* and closely related genera): evolution of life-history traits and host switches. *Molecular Phylogenetics and Evol*ution **47**, 261–273.

**Paäbo S** (1989) Ancient DNA extraction, characterization, molecular cloning and enzymatic amplification. *Proceedings of the National Academy of Sciences of the United States of America* **86**, 1939–1943.

**Perkins SL and Schall J** (2002) A molecular phylogeny of malarial parasites recovered from cytochrome *b* gene sequences. *Journal of Parasitology* **88**, 972–978.

**Petit E** (1998) Population structure and post-glacial history of the noctule bat *Nyctalus noctula* (Chiroptera, Mammalia): An analysis of European populations using mitochondrial and nuclear genetic markers. *Naturwissenschaftliche Fakultaeten, Friedrich-Alexander-Universitaet, Erlangen, Germany.*

**Ruedi M and Mayer R** (2001) Molecular systematics of bats of the genus *Myotis* (Vespertilionidae) suggests deterministic ecomorphological convergences. *Molecular Phylogenetics and Evolution* **21**, 436-448.

**Schaer J, Perkins SL, Decher J, Leendertz FH, Fahr J, Weber N and Matuschewski K** (2013) High diversity of West African bat malaria parasites and a tight link with rodent *Plasmodium* taxa. *Proceedings of the National Academy of Sciences of the United States of America* **110**, 17415–17419.

**Chumnandee et al. Supplemental Figure S1.**

**
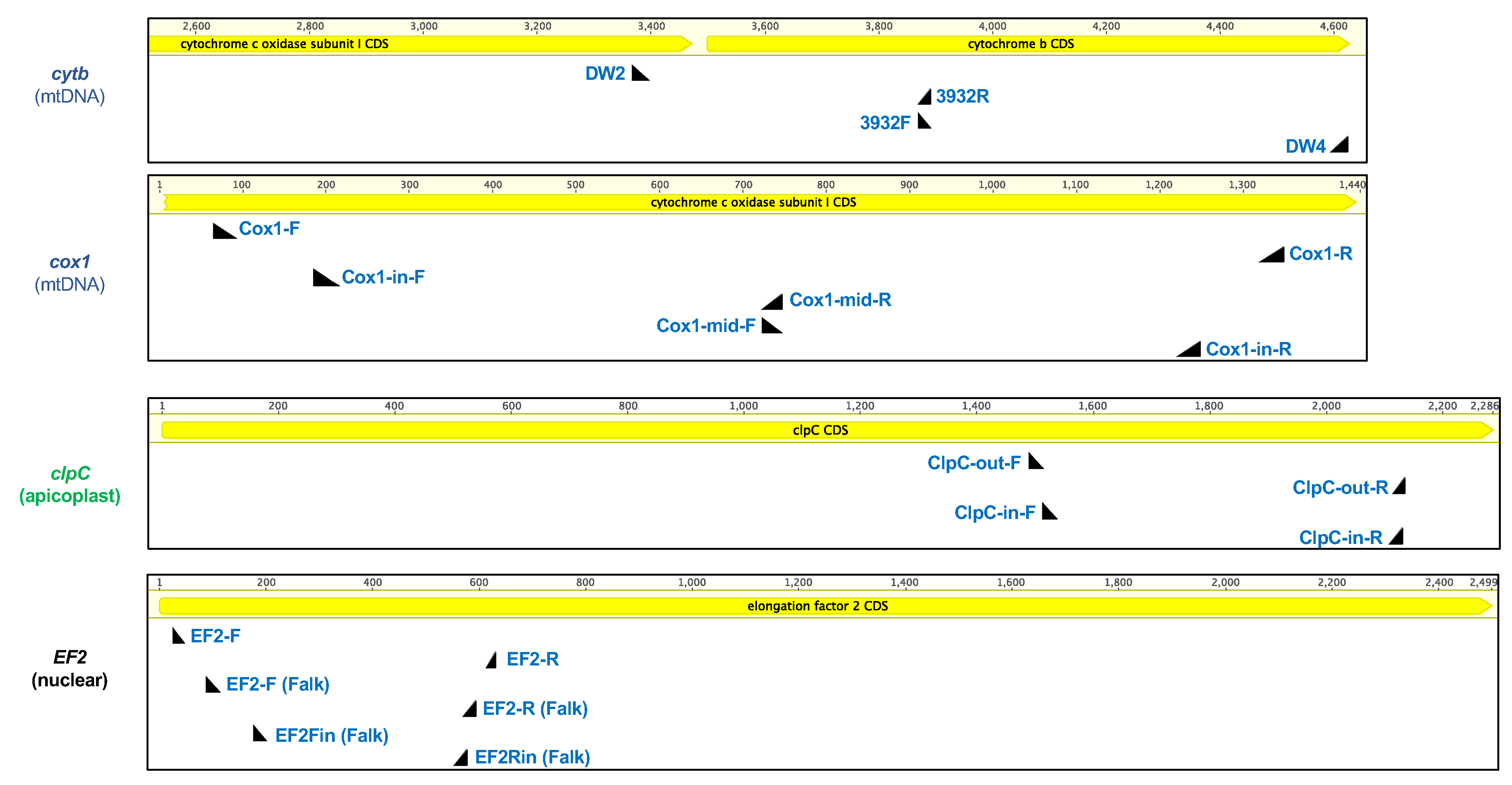
**

**Figure S1. Position of oligonucleotides in the corresponding protein-coding genes that were analyzed in the three-genome-phylogeny of *Polychromophilus* parasites.** The DW2 primer for the targeted partial cytochrome *b* sequence is located at the end of cytochrome oxidase 1, the remaining three primers are located within the cytochrome *b* gene sequence. The *cox1* primers cover almost the entire *cox1* gene, whereas the *clpC* and *EF2* primers span shorter sequences of the corresponding gene. Alignments were conducted with the software *Geneious vs.8*

**Chumnandee et al. Supplemental Table S2.**

**Table S2.** GenBank accession numbers (samples from this study highlighted in bold)

| **Parasite**  **(host group)** | **Sample (host species)** | ***Cytb*** | ***Cox1*** | ***clpC*** | ***EF2*** |
| --- | --- | --- | --- | --- | --- |
| *Leucocytozoon* (Aves) | *Leucocytozoon* sp. (2109) | EU254518 | EU254563 | EU254609 | — |
|  | *Leucocytozoon* sp. (2208) | EU254520 | EU254565 | EU254611 | — |
|  | *Leucocytozoon* sp. (P157) | EU254519 | EU254564 | EU254610 | — |
| *Parahaemoproteus* (Aves) | *Parahaemoproteus belopolskyi* | DQ451408 | EU254603 | EU254657 | — |
|  | *Parahaemoproteus coatneyi* | EU254550 | EU254595 | EU254648 | — |
|  | *Parahaemoproteus ilanpapernai* | DQ451424 | EU254591 | EU254643 | — |
| *Plasmodium*  (Aves, Squamata) | *Plasmodium floridense* | EF079654 | EF079654 | EU254620 | JN187874 |
|  | *Plasmodium gallinaceum* | AY099029 | LN835294 | AB649424 | XM_028670805 |
|  | *Plasmodium giganteum* | AY099053 | EU254577 | EU254624 | — |
|  | *Plasmodium mexicanum* | EF079653 | EF079653 | EU254619 | — |
|  | *Plasmodium relictum* | AY733090 | AY733090 | EU254633 | XM_028678091 |
| *Plasmodium* (Primates) | *Plasmodium falciparum* | DQ642845 | M76611 | DQ642846 | DQ642846 |
|  | *Plasmodium gaboni* | FJ895307 | FJ895307 | HQ842630 | XM_018788194 |
|  | *Plasmodium knowlesi* | JQ345521 | AY598141 | AF348341 | XM_002260326 |
|  | *Plasmodium malariae* | AF069624 | AB489193 | AF348342 | XM_029006894 |
|  | *Plasmodium ovale* | AF069625 | JF894415 | AY634623 | — |
|  | *Plasmodium reichenowi* | AJ251941 | AJ251941 | EU560464 | XM_012910100 |
|  | *Plasmodium vivax* | AY791692 | AY791692 | AF348344 | XM_001615828 |
|  | *Plasmodium* sp. (ex *Pan troglodytes*) | HM235391 | HM235391 | HM235147 | — |
|  | *Plasmodium* sp. (ex *Gorilla* *gorilla*) | HM235288 | HM235308 | HM235163 | XM_028684800 |
| *Plasmodium* (Rodentia) | *Plasmodium berghei* | DQ414645 | DQ414589 | DQ417612 | XM_034566733 |
|  | *Plasmodium chabaudi* | DQ414649 | DQ414593 | DQ417616 | XM_736543 |
|  | *Plasmodium vinckei* | DQ414651 | DQ414596 | DQ417619 | XM_008623839 |
|  | *Plasmodium yoelii* | AY099051 | DQ414605 | DQ417628 | LM993667 |
| *Plasmodium* (Chiroptera) | *Plasmodium cyclopsi* | KF159710 | KF159788 | KF159635 | KF159729 |
|  | *Plasmodium voltaicum* | KF159671 | KF159792 | KF159648 | — |
| *Plasmodium* (Artiodactyla) | Haemosp. sp. (ex *Anopheles gabonensis*, Gabon) | KT367860 | — | — | — |
|  | Haemosp. sp. (ex *Cephalophus dorsalis*, Gabon) | KT367821 | — | — | — |
|  | Haemosp. sp. (ex *Cephaloph. nigrifrons*, Gabon) | KT367819 | — | — | — |
|  | Haemosp. sp. (ex *Cephaloph. monticola*, Gabon) | KT367836 | — | — | — |
|  | Haemosp. sp. (ex *Anopheles marshallii*, Gabon) | KT367862 | — | — | — |
|  | Haemosp. sp. (ex *Anopheles moucheti*, Gabon) | KT367858 | — | — | — |
|  | *Plasmodium* sp. (ex *Bubalus bubalis,* Thailand) | LC090213 | LC090213 | LC090216 | — |
|  | *Plasmodium* sp. (ex *Bubalus bubalis,* Thailand) | LC090214 | LC090214 | LC090217 | — |
|  | *Plasmodium odocoilei* (ex *Odocoil. virginianus*, USA) | KU133754 | KU133756 | KU133764 | — |
|  | *Plasmodium odocoilei* (ex *Odocoil. virginianus*, USA) | KU133751 | KU133759 | KU133767 | — |
|  | *Plasmodium* sp. (ex *Capra aeg. hircus,* Zambia) | LC090215 | LC090215 | — | — |
|  | *Plasmodium* sp. (ex *Anopheles punctipennis*, USA) | KU133755 | KU133758 | KU133766 | — |
|  | *Plasmodium* sp. (ex *Anopheles punctipennis*, USA) | KU133748 | U133757 | KU133765 | — |
| *Plasmodium* (Pholidota) | Haemosp. sp. (ex pangolin, Gabon) | KT367818 | — | — | — |
| *Hepatocystis* (Primates) | *Hepatocystis* sp. (ex *Cercopithecus cephus*) | JF923760 | — | — | — |
|  | *Hepatocystis* sp. (ex *Mandrillus sphinx*) | JF923759 | — | — | — |
| *Hepatocystis* (Chiroptera) | *Hepatocystis* sp. (ex *Cynopterus brachyotis*) | EU254526 | EU254569 | EU254616 | — |
|  | *Hepatocystis* sp. (ex *Epomops buettikoferi*) | KF159779 | KF159779 | KF159612 | KF159757 |
|  | *Hepatocystis* sp. (ex *Epomophorus pusillus*) | KF159683 | KF159801 | KF159623 | KF159744 |
|  | *Hepatocystis* sp. (ex *Nanonycteris veldkampii*) | KF159698 | KF159786 | KF159631 | KF159749 |
| *Polychromophilus* (Chiroptera) | *Poly.* sp. (ex *Miniopterus minor*, NW3327, Gabon) | MK098848 | MK098850 | — | MK098856 |
|  | *Poly.* sp. (ex *Min. minor*, NW3328, Gabon) | MK098849 | MK098851 | — | MK098857 |
|  | Haemosp. sp. (ex *Min. manavi*, Madagascar) | AY762071 | — | — | — |
|  | *P. melanipherus* (ex *Min. schreibersii,* Switzerland) | KJ131271 | — | — | — |
|  | *P. melanipherus* ex *Penicillidia fulvida*, Gabon) | KU182368 | — | — | — |
|  | *P. melanipherus* (ex *Nycteribia schmidlii*, Gabon) | KU182366 | — | — | — |
|  | *P. melanipherus* (ex *Nycteribia schmidlii*, Gabon) | KU182365 | — | — | — |
|  | *P. melanipherus* (ex *Min. schreibersii,* Switzerland) | KJ131277 | — | — | — |
|  | *P. murinus* (ex *Myotis daubentonii,* Switzerland) | HM055583 | — | — | — |
|  | *P. murinus* (ex *Myotis daubentonii,* Switzerland) | HM055585 | — | — | — |
|  | *P. murinus* (ex *Myotis daubentonii,* Switzerland) | JN990712 | JN990718 | JN990723 | — |
|  | *P. murinus* (ex *Myotis daubentonii,* Switzerland) | HM055584 | — | — | — |
|  | *P. murinus* (ex *Myotis daubentonii,* Switzerland) | JN990713 | JN990719 | — | — |
|  | *P. murinus* (ex *Myotis daubentonii,* Switzerland) | HM055586 | — | — | — |
|  | *P. murinus* (ex *Myotis daubentonii,* Switzerland) | HM055587 | — | — | — |
|  | *P. murinus* (ex *Myotis daubentonii,* Switzerland) | HM055588 | — | — | — |
|  | *P. murinus* (ex *Eptesicus serotinus,* Switzerland) | HM055589 | — | — | — |
|  | *P. murinus* (ex *Myotis goudoti,* Madagascar) | MH744532 | — | — | — |
|  | *P. murinus* (ex *Myotis goudoti,* Madagascar) | MH744533 | — | — | — |
|  | *P. murinus* (ex *Myotis goudoti,* Madagascar) | MH744534 | — | — | — |
|  | *P. murinus* (ex *Myotis goudoti,* Madagascar) | MH744535 | — | — | — |
|  | *P. murinus* (ex *Myotis goudoti,* Madagascar) | MH744536 | — | — | — |
|  | *P. murinus* (ex *Penicillidia* sp.*,* Madagascar) | MH744537 | — | — | — |
|  | *P.* sp. (ex *Taphozous melanopogon*, Thailand) | MT136167 | — | — | — |
|  | *P.* sp. (ex *Myotis siligorensis*, Thailand) | MT136168 | — | — | — |
|  | *P.* sp. (ex *Kerivoula hardwickii*, Cambodia) | EF179354 | — | — | — |
|  | *P.* sp. (ex *Myotis nigricans,* Panama) | LN483038 | LN483045 | — | — |
|  | *P.* sp. (ex *Rhinolophus* sp., Bulgaria) | LN483036 | LN483046 | — | — |
|  | *P.* sp. (ex *Laephotis capensis,* Guinea) | KF159700 | — | — | — |
|  | *P.* sp. (ex *Miniopterus natalensis*, Kenya) | KT750378 | KT750454 | KT750736 | — |
|  | *P.* sp. (ex *Miniopterus natalensis*, Kenya) | KT750380 | KT750450 | KT750740 | — |
|  | *P.* sp. (ex *Myotis goudoti,* Madagascar) | AY762075 | — | — | — |
|  | *P.* sp. (ex *Miniopterus minor*, Tanzania) | KT750430 | — | — | — |
|  | *P.* sp. (ex *Pipistrellus* aff. *grandidieri*, Guinea) | KF159714 | KF159797 | KF159639 | KF159742 |
|  | ***P*. sp. (ex *Scotophilus kuhlii*, Thailand) (CC-28)** | **MT750305**  **05** | **MT750310** | — | **MT750316** |
|  | ***P.* sp. (ex *Scotophilus kuhlii*, Thailand) (CC-31)** | **MT750306** | **MT750311** | — | — |
|  | ***P*. sp. (ex *Scotophilus kuhlii*, Thailand) (CC-33)** | **MT750307** | **MT750312** | **MT750315** | **MT750317** |
|  | ***P.* sp. (ex *Scotophilus kuhlii*, Thailand) (CC-43)** | **MT750308** | **MT750313** | — | — |
|  | ***P*. sp. (ex *Scotophilus kuhlii*, Thailand) (CC-45)** | **MT750309** | **MT750314** | — | — |
|  | | | | | |
| Chiroptera | **Sample (bat)** | **ND1** | **cytb** |  | |
|  | ***Scotophilus kuhlii* (CC-28)** | **MT750322** | **MT750318** |  |  |
|  | ***Scotophilus kuhlii* (CC-31)** | — | **MT750319** |  |  |
|  | ***Scotophilus kuhlii* (CC-33)** | **MT750323** | — |  |  |
|  | ***Scotophilus kuhlii* (CC-43)** | — | **MT750320** |  |  |
|  | ***Scotophilus kuhlii* (CC-45)** | — | **MT750321** |  |  |

**Schaer *et al*., Supplemental Table S3**

**Supplemental Table S3:** Final aligned sequence (seq) length, variable and parsimony informative characters for the mitochondrial, apicoplast and nuclear alignments

| **Gene**  **(genome)** | **Aligned seq. length** | **# seqs/seqs in concat. alignment** | **Missing data** | **Variable sites** | **PICs** | **AT content** | **Gaps** |
| --- | --- | --- | --- | --- | --- | --- | --- |
| ***Cytb* (mitochondrial)** | 978 | 84/84 | 22.5% | 978 (100%) | 364 (37.2%) | 72.7% | 0% |
| ***Cox1* (mitochondrial)** | 957 | 50/84 | 45.2% | 957 (100%) | 382 (39.9%) | 70.6% | 0% |
| ***clpC***  **(apicoplast)** | 483 | 42/84 | 51.6% | 483 (100%) | 201 (41.6%) | 84.5% | 0% |
| ***Ef2***  **(nuclear)** | 375 | 21/84 | 74.5% | 375 (100%) | 96 (25.6%) | 67.1% | 0% |
| **Total/ Concatenated** | **2793** | **84** | **42.3**% | **2793 (100%)** | **1043 (37.3%)** | **73.4**% | **0%** |

Final aligned sequence length does not contain gaps, ambiguities have been converted to Ns. The concatenated alignment comprised a total length of 2793bp (including 978bp of cytochrome *b*, 957bp of cytochrome oxidase I, 483bp of the apicoplast *clpC* and 375bp of the nuclear *EF2* gene). The proportion of missing data (including data converted to Ns) was 42% (due to unavailability of published *cox1*-, *clpC*- and *EF2*-sequences for most of the *Polychromophilus* sequences). No section of the alignments was ambiguous. The proportion of variable sites was 100% and informative sites were 37%. PICs were calculated by analyzing each final alignment in the Geneious GARLI plugin (vs. 2.0, Zwickl, 2006).

**References**

**Zwickl DJ** (2006) Genetic algorithm approaches for the phylogenetic analysis of large biological sequence datasets under the maximum likelihood criterion. Ph.D. dissertation, The University of Texas, Austin, Texas, USA.
